# Molecular pathology in the diagnosis of cutaneous melanoma

**DOI:** 10.1007/s00292-026-01586-3

**Published:** 2026-09-04

**Authors:** Boštjan Luzar, Andrej Zupan, Alenka Matjašič, Jože Pižem

**Affiliations:** Institute of Pathology, Faculty of Medicine, University of Ljubljana, Korytkova 2, 1000 Ljubljana, Slovenia

**Keywords:** Cutaneous malignant melanoma, Cutaneous melanoma, WHO classification, Genetic pathways, Genetic testing, Prognosis, Kutanes Melanom, Klassifikation der WHO, Genetische Signalwege, Genetische Untersuchungen, Prognose

## Abstract

Histopathological assessment of melanocytic proliferations remains the gold standard for their correct classification. The most recent 5th edition of the World Health Organization (WHO) classification of melanocytic tumours incorporates cumulative sun damage and, in particular, molecular genetic changes to achieve this task, and recognizes nine different genetic pathways leading to melanoma development. Melanoma is therefore not a single disease genetically, but rather represents nine different well-defined clinicopathological entities that differ in terms of biological behaviour and their response to various treatment modalities. In this article, genetic pathways in the development of cutaneous melanoma are reviewed, and practical recommendations for pathologists with regards to molecular testing are provided.

## Abstract (de)

Bei melanozytischen Proliferationen stellt die histopathologische Untersuchung weiterhin den Goldstandard für deren korrekte Klassifizierung dar. Die aktuellste Klassifikation der Weltgesundheitsorganisation (World Health Organization, WHO) für melanozytische Tumoren umfasst in der 5. Auflage die kumulative Schädigung durch die Sonne und insbesondere molekulargenetische Veränderungen, um dieses Ziel zu erreichen; sie basiert auf 9 verschiedenen genetischen Signalwegen, die zur Entstehung eines Melanoms führen. Das Melanom ist daher aus genetischer Sicht nicht eine einzige Erkrankung, sondern stellt 9 verschiedene gut definierte klinisch-pathologische Entitäten dar, die sich in Bezug auf biologisches Verhalten und ihre Reaktion auf verschiedene Therapiemodalitäten unterscheiden. Im vorliegenden Beitrag wurden genetische Signalwege in der Entstehung des kutanen Melanoms zusammengefasst und praktische Empfehlungen für Pathologen unter Berücksichtigung molekularer Untersuchungen gegeben.

## Introduction

Diagnostic evaluation of melanocytic tumours is one of the most challenging areas in surgical pathology. While histopathological assessment remains the gold standard, a subset of melanocytic lesions demonstrate overlapping or ambiguous features, thus defying proper classification. In such cases, molecular diagnostic techniques provide critical complementary information. The integration of genomic data has significantly improved the accuracy of diagnosis, classification and prognostic assessment of melanocytic tumours.

Based on continuing molecular genetic research and our increasing understanding of the pathogenesis of melanocytic proliferations, the recent 5th World Health Organization (WHO) classification of melanocytic tumours combines two basic features for classifying melanocytic proliferations: a) the amount of cumulative sun damage (CSD) and b) genetic aberrations leading to melanoma development [1, 2]. Accordingly, nine different molecular pathways in the development of melanoma are recognized (Table 1).

**Table 1:** Pathways in the development of melanoma. (Table modified from [1])

Pathway	Endpoint pathway	Precursor lesion
I	*Low-CSD melanoma* Superficial spreading melanoma Melanoma in *BAP1*-inactivated melanocytoma Melanoma in WNT-activated deep penetrating/plexiform melanocytoma Melanoma in pigmented epithelioid melanocytoma	*Naevus*
II	*High-CSD melanoma*	*?*
III	*Desmoplastic melanoma*	*?*
IV	*Spitz melanoma*	*Spitz naevus*
V	*Acral lentiginous melanoma*	*Field cells*
VI	*Mucosal melanoma*	*?*
VII	*Melanoma in congenital naevus*	*Congenital naevus*
VIII	*Melanoma in blue naevus*	*Blue naevus and related benign proliferations*
IX	*Uveal melanoma*	*Uveal naevus*

*CSD* cumulative sun damage

While melanoma development in the first three pathways is causally related to ultraviolet (UV) radiation, sun damage is unrelated to melanoma pathogenesis in pathways IV to IX [1]. Not all melanomas develop from a precursor melanocytic naevus, and some melanomas, such as acral melanomas, arise from field cells, defined as basal melanocytes with normal or slightly enlarged nuclei sharing genetic features with adjacent melanoma [3]. Melanoma is not a single disease. Rather, nine pathways of melanoma development suggest nine different diseases with distinctive genetic, clinical and morphological features but also differing significantly in terms of biological behaviour, prognosis and treatment [2].

Herein, each of the genetic pathways for development of cutaneous melanoma is reviewed.

## Pathway I: low-cumulative-sun-damage melanoma

### Background

Low-CSD melanoma is a variant of melanoma arising in low or intermittently sun-damaged skin [1]. Four main variants are recognized [1]:

- superficial spreading melanoma, representing a prototype of low-CSD melanoma;
- *BAP1*-inactivated melanoma;
- WNT-activated deep-penetrating/plexiform melanoma and
- malignant pigmented epithelioid melanocytoma (PEM).

While SSM develops from precursor melanocytic naevi in about 30% of cases, including common acquired and congenital melanocytic naevi, other low-CSD melanoma variants typically develop from pre-existent melanocytomas [4]. Following driver genetic aberrations, usually activating mutations in the *BRAF* or *NRAS* genes, secondary genetic aberrations determine the genetic pathway and morphological features of a precursor intermediate-grade melanocytic proliferation (melanocytoma): *BAP1*-inactivated melanocytoma, WNT-activated melanocytoma or PEM [1]. In addition to superficial spreading melanoma, *BAP1*-inactivated melanoma and WNT-activated melanoma are reviewed in the current article.

### Superficial spreading melanoma

#### Definition

**Fig. 1:**
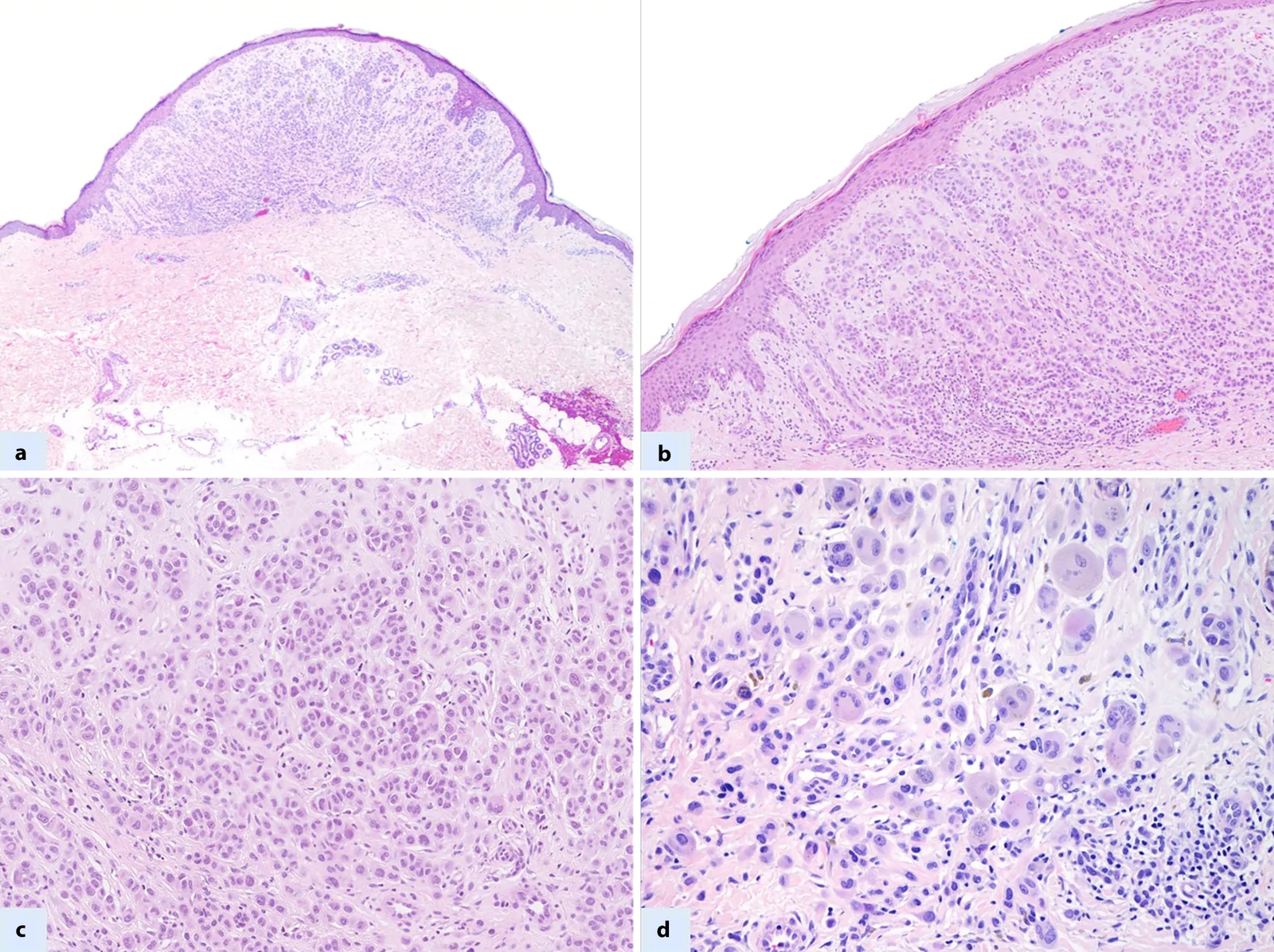
Superficial spreading melanoma on the left calf in a 51-year-old female (regarded as an atypical Spitz tumour on haematoxylin and eosin stain). DNA next-generation sequencing: *NRAS* exon 3 c.182A>G p.(Gln61Arg)(Q61R), *NF1* c.1721+2T>C in the splice site of exon 15 (intron 15-16), *ALK* p.(Trp1320Ter)(W1320*) in exon 27, deletion of *CDKN2B* and *CDKN2B*. **a** Low-power magnification of a fairly symmetrical predominantly intradermal melanocytic proliferation. **b** Subtle lentiginous proliferation with focal upward migration of melanocytes can be seen in this picture. **c** Melanocytes have epithelioid (spitzoid) morphology. **d** Notice severe pleomorphism of tumour cells on higher magnification

Superficial spreading melanoma (SSM) is a variant of melanoma arising in low or intermittently sun-damaged skin ([1]; Fig. 1).

#### Genetic landscape

The most common driver genetic aberration in SSM is a *BRAFV600E* mutation, followed by a *NRASQ61R* mutation (70% and 20%, respectively.) [3–5]. Progression from melanocytic naevus to melanoma in situ is achieved by an accumulation of additional genetic changes, including heterozygous *CDKN2* loss and *TERT *promoter mutation [6]. Development of invasive melanoma is associated with homozygous loss of *CDKN2,* leading to the loss of cell cycle regulation, with additional mutations in the tumour suppressor genes *TP53* and *PTEN *[6]. According to this scenario, *BRAF* mutations activate the MAPK pathway, while *PTEN* loss leads to PI3 kinase pathway activation [7]. On the other hand, *NRAS* mutation alone activates both the MAPK pathway and the PI3 pathway simultaneously, without the need for *PTEN* loss [7].

#### Prognosis

A recent analysis encompassing over 13,000 patients revealed the cancer-specific survival of SSM patients at 1, 3 and 5 years to be 0.91, 0.92 and 0.89, respectively [8]. Furthermore, independent predictors of overall and cancer-specific survival in SSM were age, sex, tumour site, American Joint Committee on Cancer T stage and first primary melanoma [8]. Histological parameters related to prognosis include tumour thickness, mitotic activity, ulceration, lymphovascular invasion and microscopic satellites [1].

### *BAP1*-inactivated melanoma

#### Definition

**Fig. 2:**
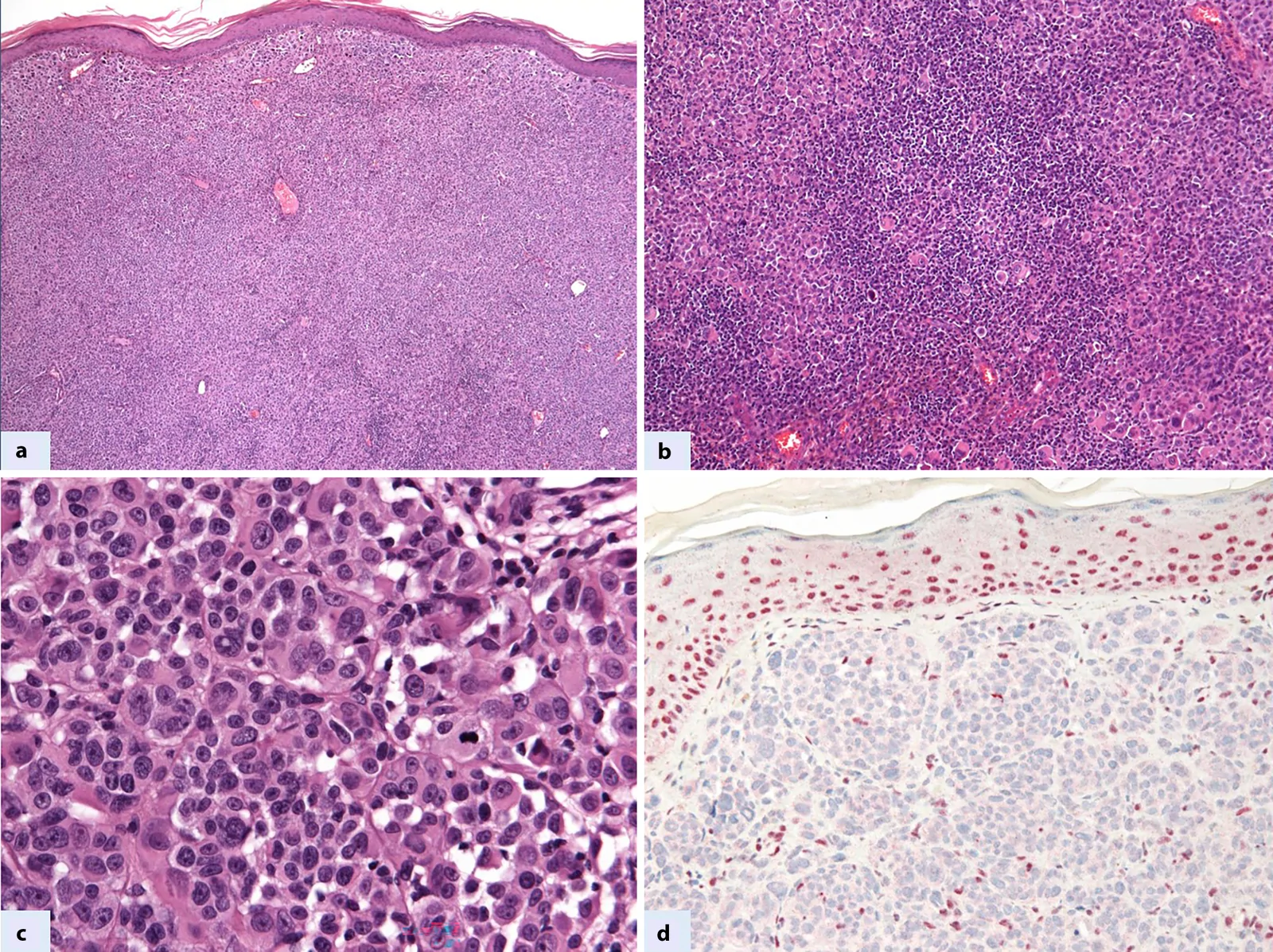
*BAP1*-inactivated melanoma on the back in a 24-year-old female with BAP1-related tumour predisposition syndrome. **a** Low-power magnification depicting a sheet-like proliferation of melanocytes in the dermis. **b** Brisk inflammatory cell infiltrate is present on the background of melanocytic proliferation featuring epithelioid melanocytes. **c** High-power magnification demonstrating severe cytological atypia and brisk mitotic activity. **d** BAP1 immunohistochemistry with a negative reaction in the melanocytic nuclei. Note the positive internal control in keratinocytes and lymphocytes

This melanoma variant results from progression of *BAP1*-incativated melanocytoma, either in a sporadic setting or on the background of BAP1-related tumour predisposition syndrome [9]. By definition, all tumours are characterised by *BAP1* inactivation, representing a secondary pathogenetic variant in combination with a *BRAFV600E* mutation as the driver genetic change and, much more infrequently, with an *NRASQ61R* driver mutation occurring in a mutually exclusive pattern ([10–12]; Fig. 2).

#### Genetic landscape

*BAP1*-inactivated melanoma develops in a stepwise molecular progression model from a melanocytic naevus. According to this model, the driver genetic aberrations activating the MAPK pathway represent the first genetic hit leading to the development of a melanocytic naevus, usually a common acquired naevus [9, 10]. Activating *BRAFV600E* mutations predominate by far, followed by *NRASQ61R* activating mutations, while *RAF1* fusion as a driver has been reported only exceptionally [10, 12–14]. A secondary genetic hit is represented by inactivation of the *BAP1* wildtype allele, leading to biallelic *BAP1* inactivation, also confirmed by deletion of 3p, the location of the *BAP1* gene [15]. This genetic aberration is reflected in the appearance of a second melanocytic clone distinguished by an epithelioid (spitzoid) morphology—*BAP1*-inactivated melanocytoma. The third hit is represented by further genetic progression, including *TERT* promoter mutation, and usually in excess of three copy number variations, leading to *BAP1*-inactivated melanoma [16].

#### Prognosis

Due to the limited number of *BAP1*-inactivated melanomas published in the literature, the prognosis remains uncertain. While some studies have suggested much more indolent biological behaviour, despite high tumour thickness and high mitotic activity in comparison to thickness-matched conventional melanomas [16, 17], some *BAP1*-inactivated melanomas follow an aggressive clinical course, with rapid early systemic dissemination within the first 2 years after diagnosis [18].

### WNT-activated deep-penetrating/plexiform melanoma

#### Definition

**Fig. 3:**
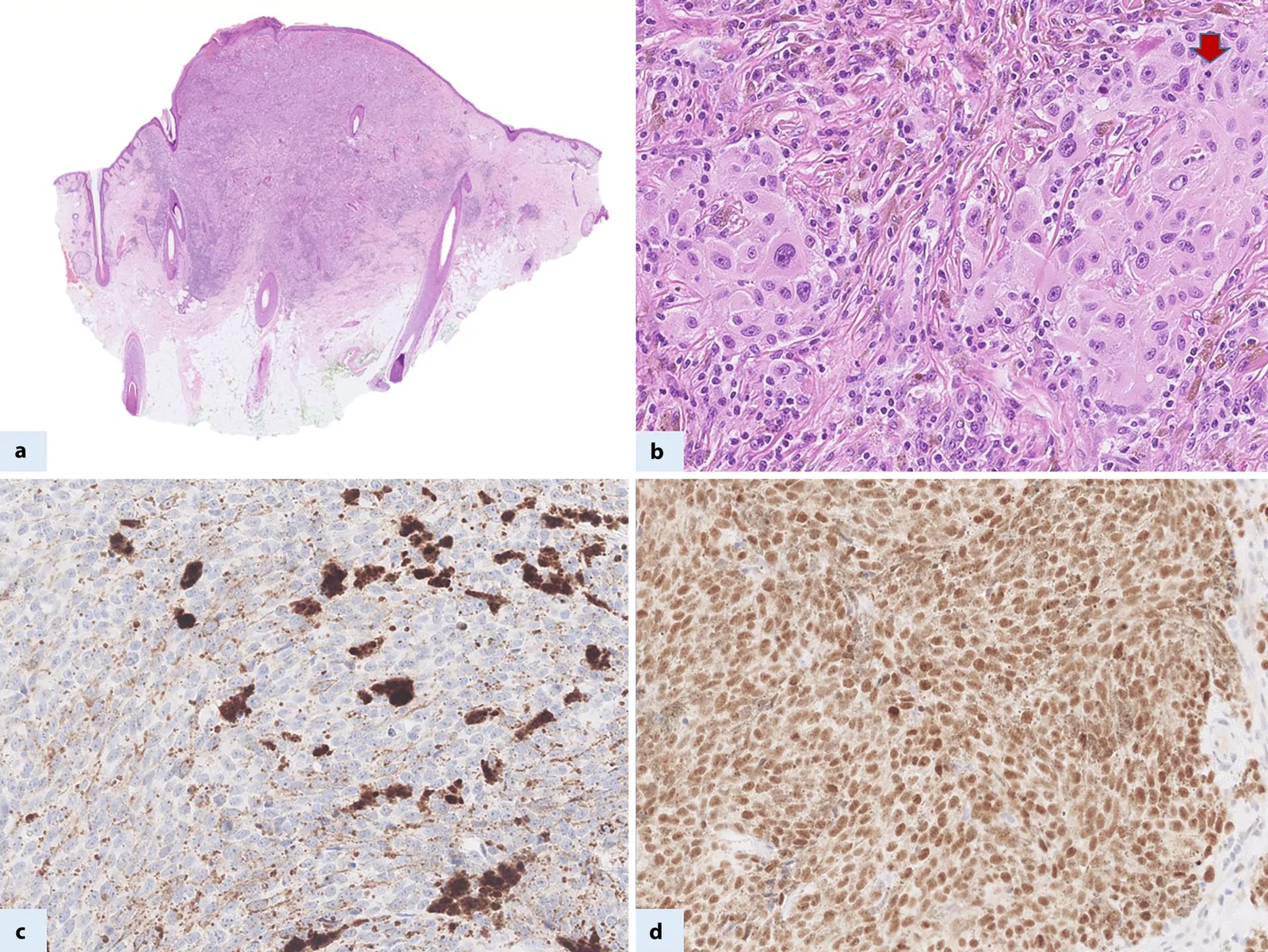
WNT-activated deep-penetrating/plexiform melanoma on the parietal scalp in a 72-year-old female. DNA next-generation sequencing: *BRAFV600R* p.(Val600Arg); *TERT* promoter c.146C>T; *CTNNB1* c.122_123delinTT p.(Thr41II)(T41L); *ARID2* c.4231C>T p.(Gln1411Ter)(Q1411*); *RAC1* c.85C>T p.(Pro29Ser)(P29S); *SF3B1* c.2939A>C p.(Glu980Ala)(E980A); deletion of GNAQ at 9p21.2. **a** Low-power magnification highlighting a nodular intradermal proliferation, ulcerated on the surface. **b** Highly atypical tumour cells forming nests. Note mitotic activity (arrow). **c** Complete absence of p16 immunopositivity. **d** Tumour cells are diffusely PRAME positive (> 75% of tumour cells)

WNT-activated deep-penetrating/plexiform melanoma (WNT-M) is a variant of melanoma characterised genetically by combined activation of the MAPK pathway, acting as a driver genetic aberration, followed by activation of a WNT signalling pathway [19]. The majority of WNT‑M develop from a pre-existent melanocytic naevus through an intermediate stage designated a WNT-activated melanocytoma ([19]; Fig. 3).

#### Genetic landscape

The driver activating mutations in the MAPK kinase pathway, usually *BRAFV600E, NRASQ61R* or *MAP2K1,* and secondary pathogenetic variants involving the Wnt/β-catenin pathway, either *CTNNB1* or *APC, *are distinctive of this melanoma variant [19]. Progression to WNT‑M is achieved by accumulating additional genetic aberrations, most commonly pathogenetic variants in the *TERT* promoter, *CDKN2A* gene and protein subunits of the SWI/SNF complex [20, 21].

#### Prognosis

Few examples of WNT‑M have been published in the literature [19, 21, 22]. Spread to the local lymph nodes has been reported in a subset of tumours [20–22]. Nevertheless, wide systemic dissemination with death from the disease appears to be an exception [19].

## Pathway II: high-cumulative-sun-damage melanoma/lentigo maligna melanoma

### Definition

**Fig. 4:**
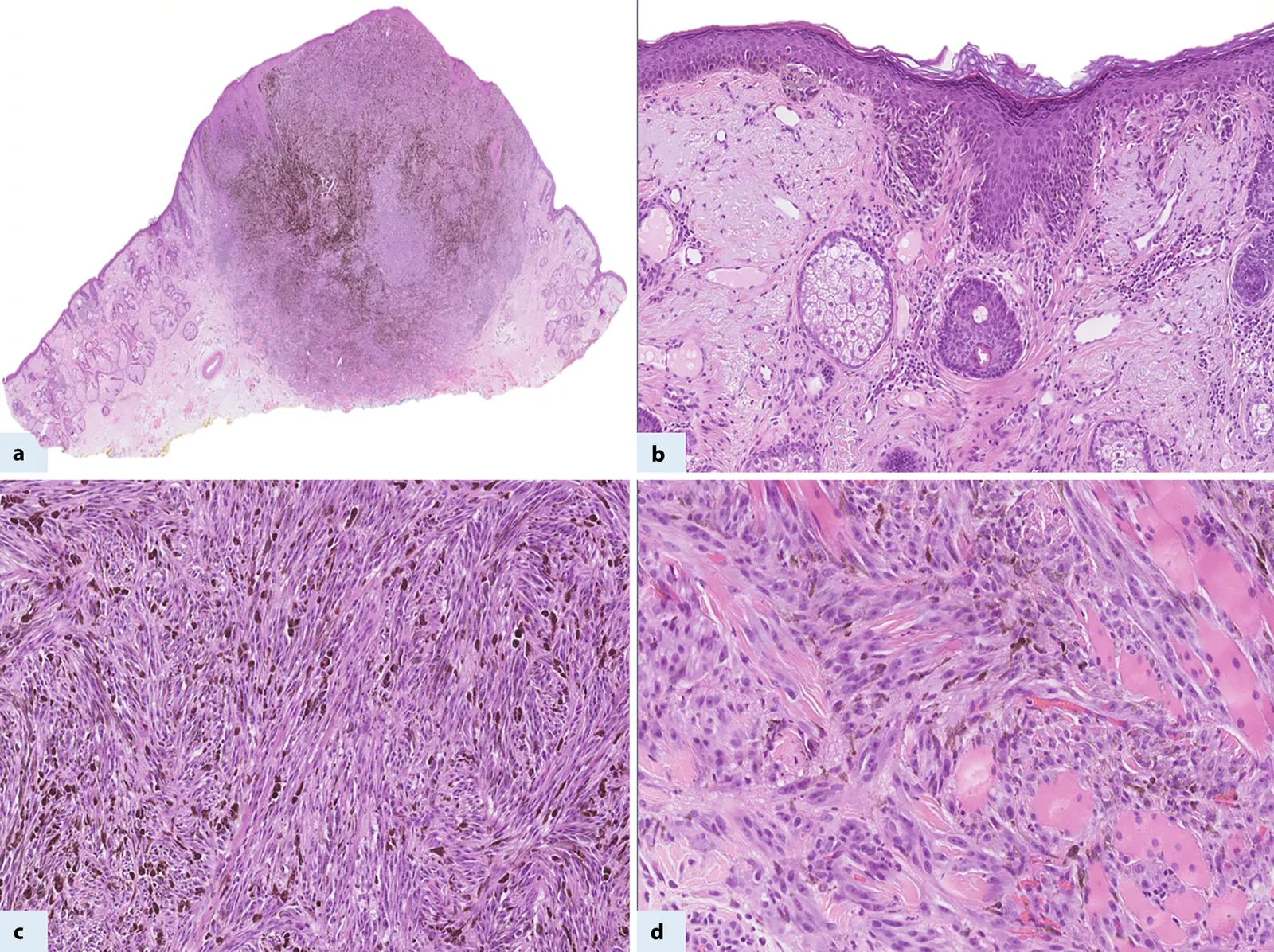
Lentigo maligna melanoma on the nose in a 55-year-old male. DNA next-generation sequencing: *NF1* exon 18 c.2041 C>T p.(Arg681Ter)(R681*); *TERT* promoter c.124C>T; *TP53* exon 10 c.1024C>T p.(Arg342Ter)(R342*); amplification of *BRAF*, *MET* and *RREB*; deletion of genes *BAP1*, *CTNNB1*, *MITF*, *PIK3CA* and *TP53*; deletion of 3p21 and 17p13.1; loss of heterozygosity at 17q11.2. **a** Irregularly pigmented infiltrative and somewhat nodular proliferation in the dermis can be appreciated in this photo. **b** A lentigo maligna (melanoma in situ) component can easily be identified at the periphery of the main tumour mass. Note prominent solar elastosis. **c** Invasive component consists of heavily pigmented spindled melanocytes. **d** In some areas, more epithelioid tumour cells are seen infiltrating the skeletal muscle

High-CSD melanoma is a variant of melanoma arising on severely sun-damaged skin. Lentigo maligna melanoma (LMM) [1] typically arises from a pre-existent melanoma in situ, also designated lentigo maligna (LM; Fig. 4).

### Genetic landscape

The mutational burden in LM/LMM is especially high due to chronic cumulative exposure to UV light, with corresponding UV signature mutations [23]. The genetic profile of LMM differs significantly from that of low-CSD melanoma in terms of the frequencies and types of driver genetic aberrations. While *BRAF* mutations are present in up to 80% of low-CSD melanomas, they are detected in 10%–20% of LMMs, with a predominance of a non-*BRAFV600E* mutations, in particular *BRAFV600K* mutations [24]. *NRAS* mutations are drivers in up to 14% of LMMs [25]. *KIT *mutations occur in less than 2% of low-CSD melanomas and are present in 25%–28% of LMM [26, 27]. *NF1-*inactivating mutations have been detected in 46% of LMM expressing wildtype *BRAF* and *RAS *[28]. Secondary genetic changes include inactivation of the tumour suppressor genes *TP53, CDKN2A, CCND1 *and *ARID2* as well as recurrent *TERT* promoter mutations [4, 28].

### Prognosis

Complete surgical excision with sufficient tumour-free margins is crucial for preventing local recurrence. In a study analysing over 1500 cases of LM, a 12-mm clinical margin was needed to achieve a 97% clearance rate in the head and neck area [29]. In cases of LMM, the prognosis appears to be similar to that of other invasive melanomas adjusted for Breslow thickness [1]. Furthermore, mitotic count, ulceration, the presence of lymphovascular invasion and microscopic satellites are likely to influence the prognosis of LMM [1].

## Pathway III: desmoplastic melanoma

### Definition

**Fig. 5:**
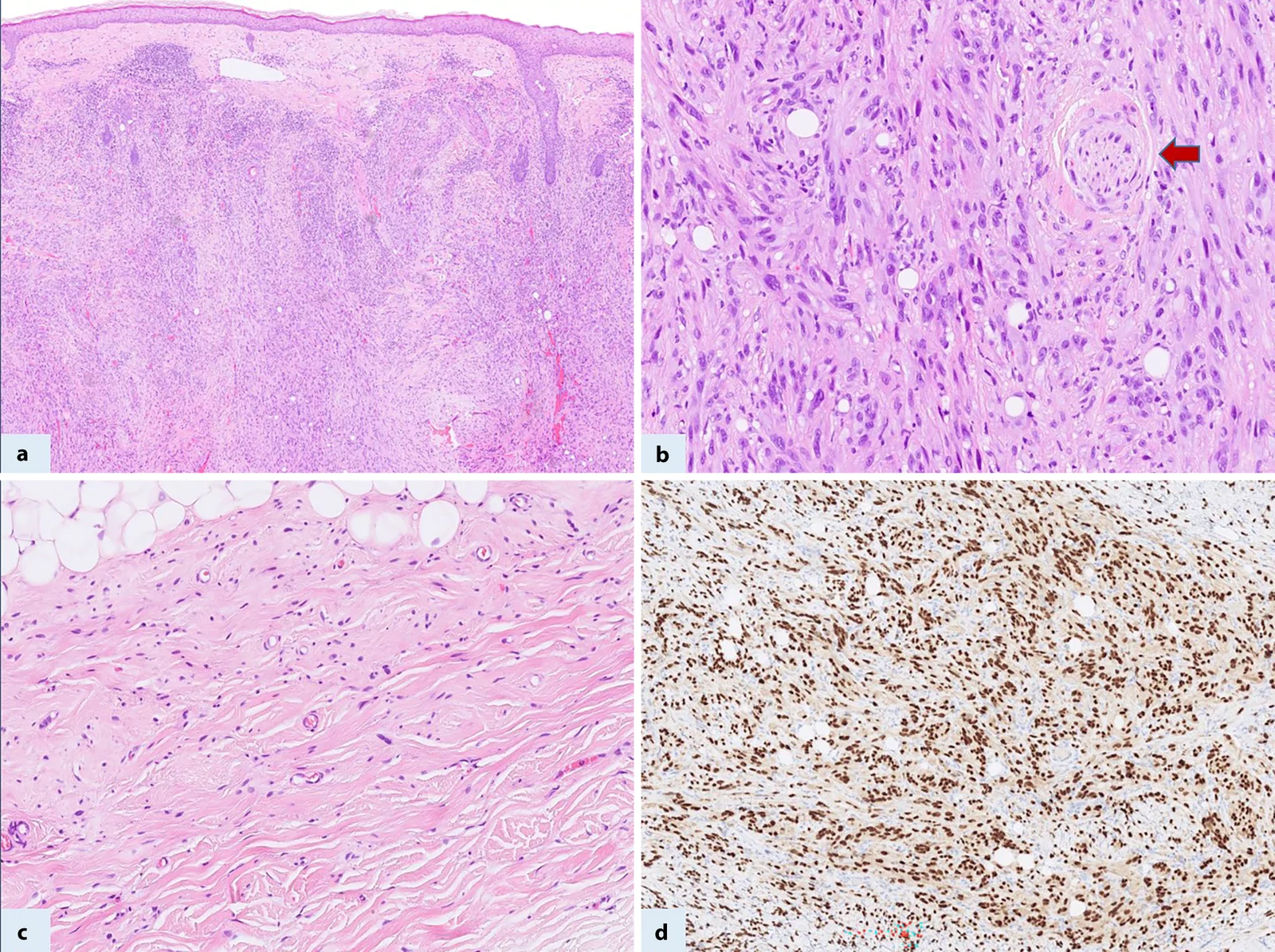
Desmoplastic melanoma (mixed variant) on the scalp in a 68-year-old male. DNA next-generation sequencing: *NF1* exon 9 c.983_984delGT p.(Cys328Ter)(C328*); *NF1* exon 21 c.2557C>T p.(Gln853Ter)(Q853*); *EGFR* exon 19 c.2197C>T p.(Pro733Ser); amplification of chromosomes 15 and 17; heterozygous deletion of chromosome 9 and 1p. **a** Low-power magnification of a highly infiltrative spindle cell proliferation with involvement of the subcutis. Note the absence on the intraepidermal tumour component. **b** Proliferation of moderately atypical spindle cells with neurotropism (arrow). **c** Pure desmoplastic melanoma can be notoriously difficult to diagnose—pay attention to cells with hyperchromatic nuclei. **d** Diffuse SOX10 positivity is a characteristic feature in desmoplastic melanoma

Desmoplastic melanoma (DM) is a subtype of melanoma composed of spindled fibroblast-like melanocytes with tumour cells separated by collagenous stroma ([1, 2, 30]; Fig. 5).

### Genetic landscape

Genomic changes in DM are very different to those of conventional melanoma. Namely, DM ranks among the most highly mutated cancers [31]. The majority of mutations (nearly 90%) represent C>T transitions causally related to UV radiation as a dominant mutagen [31]. Characteristically, DM generally lacks the MAPK pathway mutations seen in classical pathway I melanoma, such as *BRAF* p.V600E and *NRAS* p.Q61R/K mutations [31, 32]. Rather, it often harbours biallelic inactivation of *NF1* (45%–90%), leading to a loss of NF1 protein and thus resulting in chronic hyperactivation of the RAS-RAF-MEK pathway, combined with:

- genetic aberrations leading to activation of the RTK-RAS-MAPK/PIK3 signalling pathway (73%), including copy number alterations of *MAP3K1, EGFR, MET* and *SOS2*, and mutations of *CBL, ERBB2, MAP2K1, BRAF *other than p.V600E, *PTPN11, RAC1, NRAS* and *PIK3CA;*
- recurrent mutations in the p53 and Rb pathways (up to 66% and 71%, respectively), mostly inactivation of *TP53, RB1 *and *CDKN2A*; and
- inactivating mutations of the SWI/SNF chromatin remodelling complex (22%), such as *ARID2* and *ARID1A* [28, 31–34].

In addition, while over 90% of DM harbour *TERT* activation by either promoter mutation or amplification, about 15% of DM feature recurrent *NFKBIE* promoter mutations, with the significance of the latter yet to be elucidated [31].

### Prognosis

In comparison to conventional melanoma, DM is a locally aggressive tumour with a high incidence of local recurrence (especially in cases with marginal excision) and a low incidence of regional metastases [35, 36]. No significant differences in melanoma-specific mortality have been observed between pure DM and conventional melanoma, despite a mean tumour depth of 4.5 mm for pure DM and 2.1 mm for conventional melanoma [37]. Nevertheless, mixed DM has significantly higher 2‑ and 5‑year melanoma-specific mortality than pure DM [37]. Furthermore, pure DM is associated with less regional lymph node metastasis than the mixed variant (0%–18.2% and 8.5%–25%, respectively, across different studies) [37]. The prognosis of DM is influenced by tumour thickness, mitotic rate, increasing age and male sex [38].

## Pathway IV: Spitz melanoma

### Definition

Spitz melanoma is a subtype of melanoma characterized histologically by Spitz morphology of tumour cells (e.g. large epithelioid/spindled melanocytes) and genetic alterations defining a Spitz melanocytic linage [1, 39, 40].

Several studies have clearly demonstrated that morphological features alone are an unreliable predictor of the Spitz melanocytic lineage [41, 42]. Relying on morphology alone will therefore ultimately lead to misdiagnosing a substantial proportion of spitzoid melanomas, usually MAPK-pathway-driven melanomas with a much worse prognosis but which are amenable to specific treatment as Spitz melanomas [41, 42].

For practical purposes, establishing a diagnosis of Spitz melanoma essentially requires

**Fig. 6:**
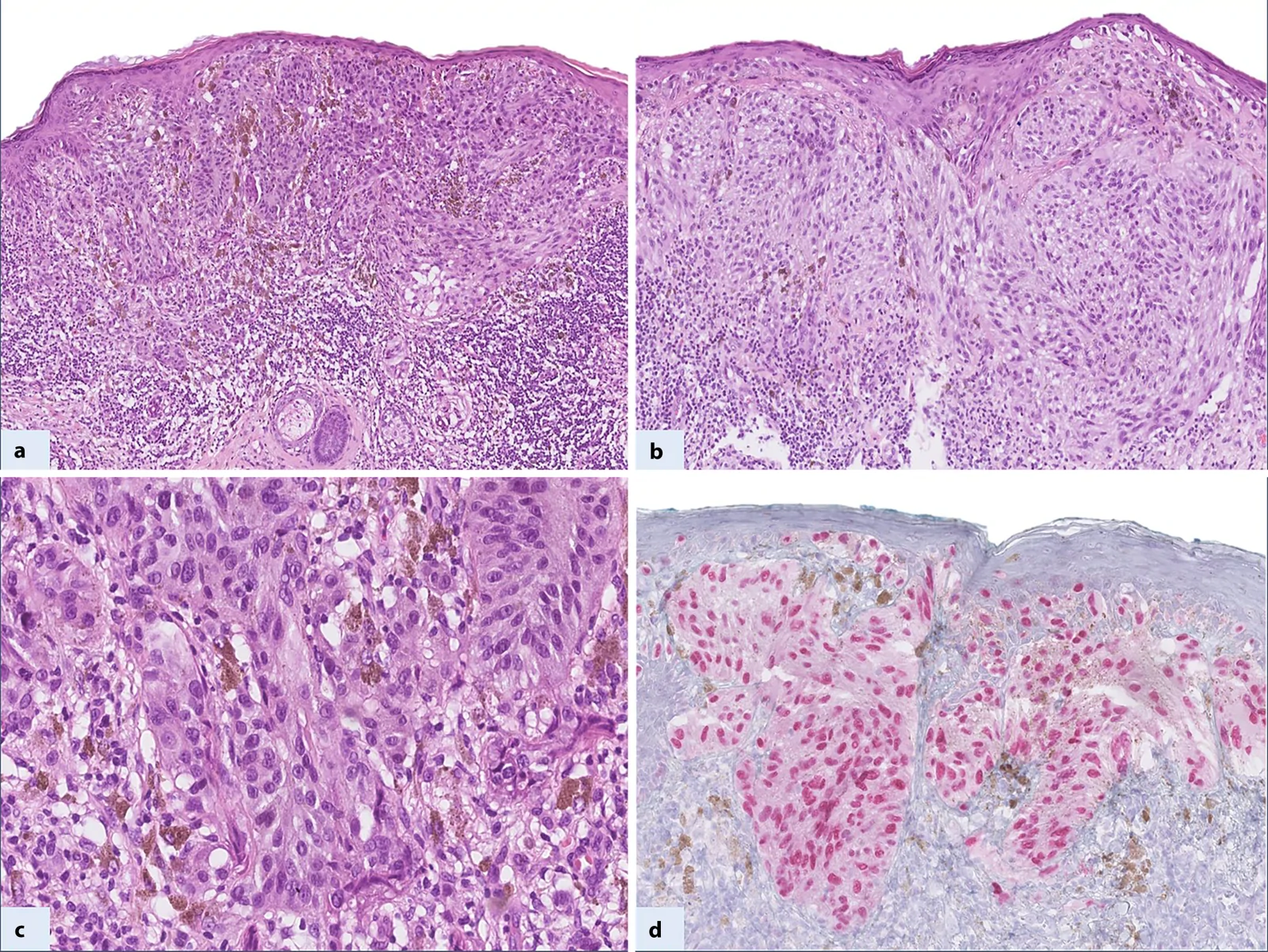
Spitz melanoma with *HRAS* mutation on the left ear in a 64-year-old female. DNA next-generation sequencing: *HRAS* exon 2 c.37G>C p.(Gly13Arg)(G13R), *TERT* promoter c.146C>T, *ARID2* exon 15 c.3109C>T p.(Gln1037Ter)(Q1037*), *HRAS* amplification at 11p15.5, deletion of *CCND1 *and loss of heterozygosity at 12q12. **a** Low-power magnification of an asymmetrical compound melanocytic proliferation with irregular pigmentation and immune host response in the dermis. **b** Melanocytes in the dermis grow in expansile nests. **c** Epithelioid or Spitz morphology is readily apparent. **d** Immunohistochemistry for PRAME with diffuse 4+ (> 75% of tumour cells) positivity

- confirmation of a Spitz genetic pathway (e.g. driver genetic aberration) by immunohistochemistry, FISH and/or RNA/DNA next-generation sequencing and
- detecting additional genetic changes (e.g., pathogenetic variants) associated with genetic progression to melanoma by next-generation sequencing ([1, 41, 43]; Fig. 6).

### Genetic landscape

Molecular aberrations in Spitz melanocytic proliferations can be divided into two main groups according to the driver genetic change:

- 11p amplification/*HRAS* mutation, detected in about 20% of Spitz melanocytic proliferations, and
- kinase fusions, present in about 50% of Spitz melanocytic proliferations, further subdivided into tyrosine kinase fusions (*ALK, ROS1, NTRK1, NTRK2, NTRK3, RET, MET*) and serine/threonine kinase fusions (*MAP3K8, BRAF*).

The abovementioned driver genetic aberrations can be detected along the whole biological spectrum of Spitz melanocytic proliferations, including Spitz naevi, atypical Spitz tumours/melanocytomas and Spitz melanomas. Importantly, however, the driver genetic aberrations are mutually exclusive in a given Spitz melanocytic proliferation [39]. Furthermore, additional genetic changes are obligatory for Spitz melanoma development [39, 44]. Spitz melanomas should have, in addition to the defining driver genetic aberration, three or more chromosomal abnormalities, often associated with *TERT* promoter and *PTEN* mutations [1, 39, 44].

### Prognosis

The prognosis of Spitz melanomas is influenced by underlying genetic abnormalities, including the driver genetic changes. Spitz melanomas with an *HRAS* mutation and those with tyrosine kinase fusions generally follow an indolent clinical course, on occasion associated with deposits in the regional lymph node(s), while systemic dissemination and death from the disease has not been reported to date [44, 45]. In contrast, fatal Spitz melanomas have only been associated with tumours featuring serine/threonine kinase fusions like a *BRAF* or *MAP3K8* fusion [45].

## Pathway V: acral melanoma

### Definition

**Fig. 7:**
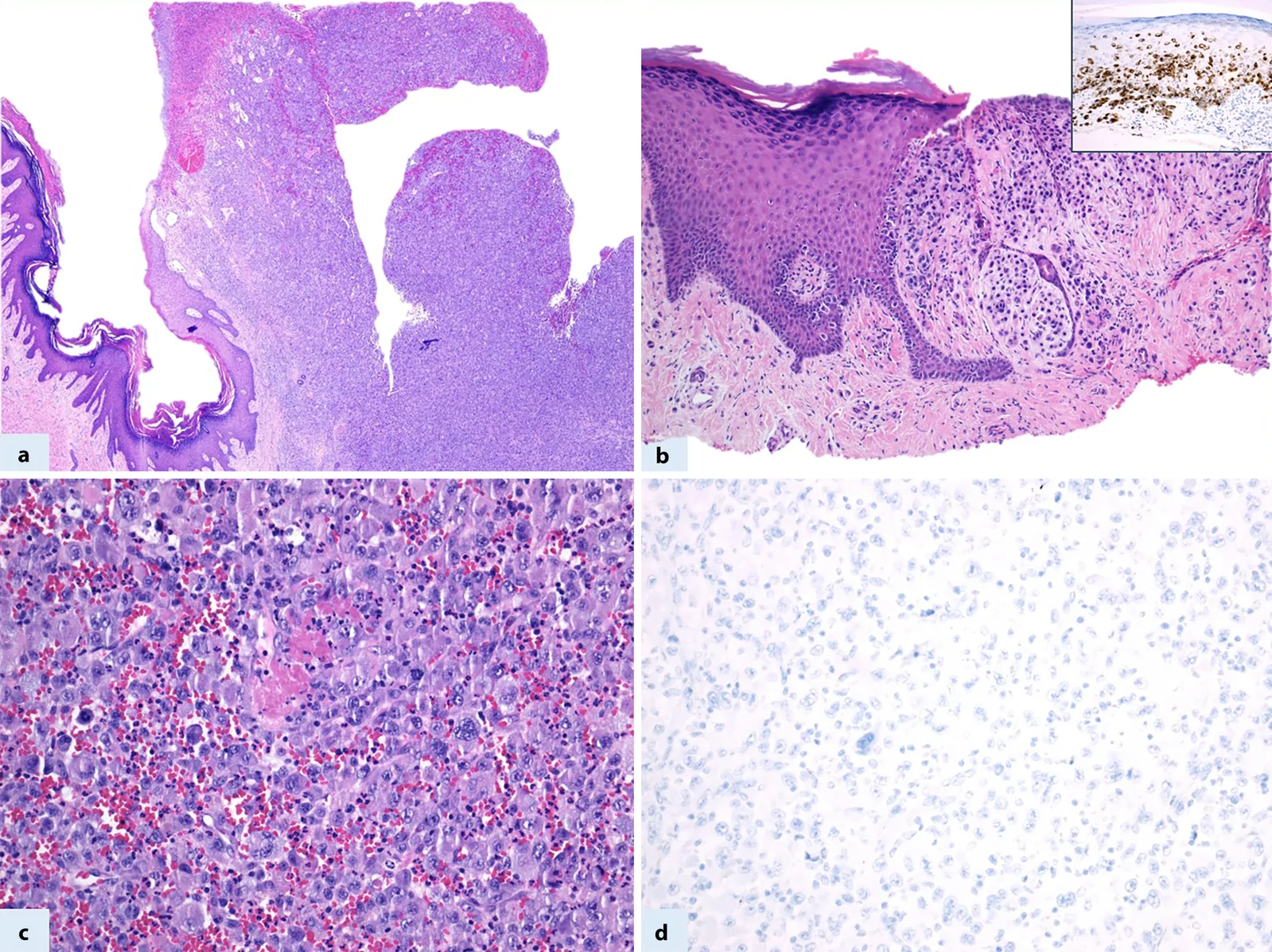
Dedifferentiated acral melanoma on a right toe in an 82-year-old female. **a** Low-power magnification of an ulcerated tumour on acral skin. **b** Area of acral skin with both melanoma in situ and invasive melanoma. Inset picture showing diffuse melan A positivity of tumour cells. **c** Dermal component featuring an undifferentiated pleomorphic tumour with brisk mitotic activity. **d** The majority of the dermal component lacks melan A staining. All other melanocytic markers, including S100, SOX10, HMB45, tyrosinase and MITF, were also negative in this component (data not shown)

Acral melanoma is a melanoma variant arising on the palms and soles, nail apparatus, and on glabrous (non-hair bearing) skin of the volar surfaces of the fingers and toes [1]. Melanomas developing on the dorsal aspects of these sites are often related to cumulative sun damage and are genetically distinct from acral melanomas as defined above. This variant of melanoma does not arise from a pre-existing melanocytic naevus ([46]; Fig. 7).

### Genetic landscape

Acral melanoma is characterized by the lack of an UV-radiation-induced mutational signature, low mutational burden, and a high frequency/burden of both somatic structural mutations and copy number alterations [47, 48]. The latter include the presence of multiple narrow high-level amplifications, deep deletions, and complex intra- and inter-chromosomal rearrangements [48]. Chromothripsis and chromoplexy (so-called hailstorms) appear to be the mechanisms for a high frequency of complex rearrangements and represent a unique feature of acral melanomas [49].

Approximately 50% of acral melanomas lack mutations in the main melanoma driver genes, such as *BRAF, NRAS* and *NF1* (so-called triple-negative melanomas) [50]. A recent study on 212 acral melanomas detected *NRAS* (21.1%), *BRAF* (18.3%) and *KIT* (9.0%) mutations as the most common in this type of melanoma [51]. In addition, copy number variations with deletions of the tumour suppressor genes *CDKN2A* and *CDKN2B* were present in more than 20% of tumours (24.2% and 21.4%, respectively), followed by amplification of oncogenes, including *CDK4* (19.9%), *CCND1* (19.1%) and *TERT* (19.9%) [51]. These gene amplifications represent important drivers in the development of acral melanoma. The co-occurrence mutations between *PTPRT*/*NF1* and *TERT*/*KRAS* may prove to be unique to acral melanoma [51]. *NAB2* mutations have only been detected in metastatic tumours [51].

A recent genetic model for the development of acral melanoma proposed an inverted order of genetic aberrations in comparison to the canonical evolution of classic melanoma, with *TERT* activation and hailstorm formation representing early genetic events, already present in individual melanocytes (so-called field cells, occult melanoma in situ), while MAP-kinase pathway mutations typically develop later in the process [48]. The study also confirmed a frequent occurrence of recurring changes on chromosomes 5, 11, 12 and 22 in acral melanoma (78.4%), including clustered copy number transitions with focal amplifications [48].

### Prognosis

Acral melanoma is an aggressive variant of melanoma with significantly lower melanoma-specific survival than conventional melanoma, mainly due to delayed diagnosis (5-year survival of 80.6% and 93%, respectively) [52].

## Pathway VII: melanoma arising in congenital naevus

### Definition

**Fig. 8:**
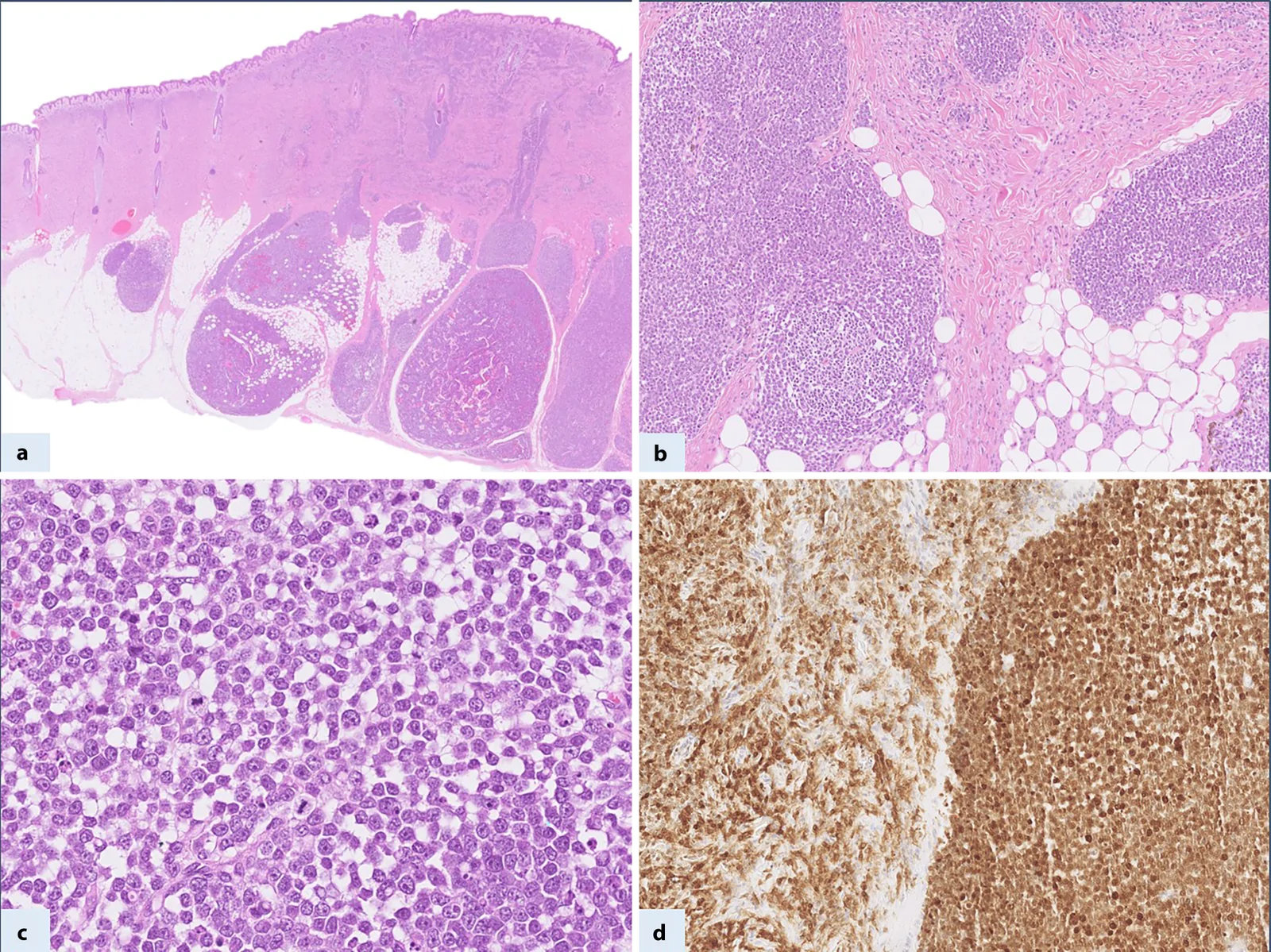
Melanoma in a giant congenital naevus on the back in an 1‑year-old girl. The patient died of lung metastases 18 months after initial diagnosis. **a** Low-power magnification depicting a congenital melanocytic naevus with extension into the subcutis, associated with multinodular proliferation in the subcutaneous fat. **b** Abrupt transition between the two melanocytic components (naevus in the middle). **c** Poorly differentiated round cell component of melanoma with brisk mitotic activity. **d** Both benign and malignant components are diffusely S100 positive

This variant of melanoma develops on the background of a congenital melanocytic naevus, defined as a melanocytic naevus/proliferation present at birth or becoming apparent during the first weeks of life ([1]; Fig. 8).

### Genetic landscape

Activating *NRAS* mutations in codon 61 (predominantly *NRASQ61K*/*L*/*R*) represent the most common driver genetic change in giant congenital melanocytic naevi [53, 54]. Interestingly, in small/intermediate congenital melanocytic naevi, *BRAFV600E *driver mutations predominate [55]. In contrast, less than 1% of giant congenital naevi harbour *BRAFV600E *activating mutations [56]. Exceptionally, different types of fusions, including *BRAF, RAF1, PRKC *and *ALK*, fusions have been reported as drivers in giant congenital melanocytic naevi [57–59]. Progression to melanoma is achieved by accumulating additional genetic aberrations involving *TP53, CDKN2A, KIT, KDR, MC1R, MAP2K1, MITF* and *TERT* promoter mutations [54]. By comparative genomic hybridization, complex chromosomal aberrations, including numerous gains and losses of arms or segments of different chromosomes, are typical of melanoma [60, 61].

### Prognosis

Melanoma arising in giant congenital melanoma represents a particularly aggressive subset of paediatric melanoma, with rapid disease progression despite multimodal treatment [62].

## Pathway VIII: melanoma arising in a blue naevus

### Definition

**Fig. 9:**
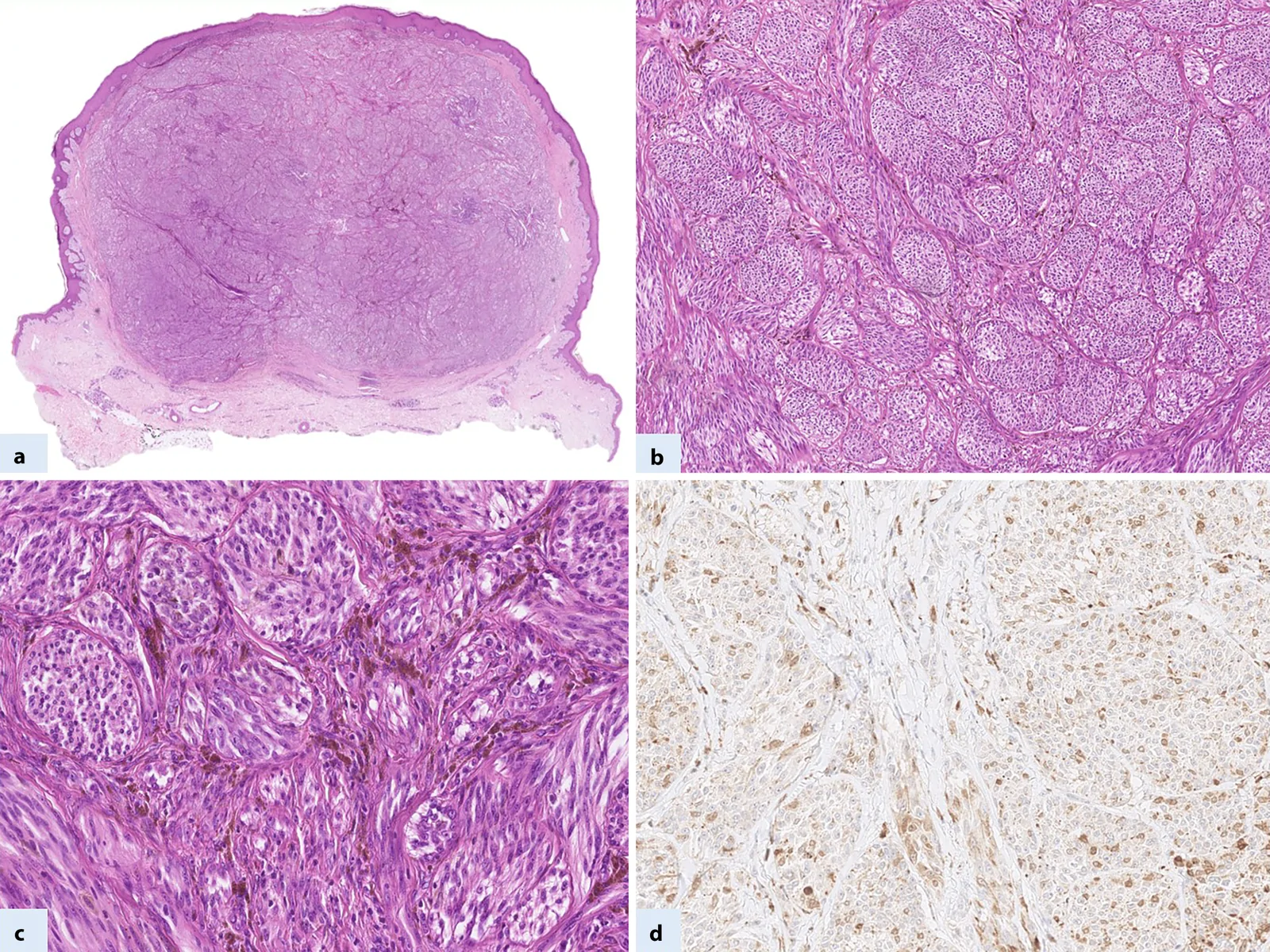
Malignant blue naevus on the right thigh in an 80-year-old male. DNA next-generation sequencing: *GNA11* exon 5 c.626A>T p.(Gln209Leu)(Q209L); *KRAS* exon 3 c.182A>G p.(Gln61Arg)(Q61R); *TERT* promoter c.124CC>TT; *EIF1AX *exon 1 c.12T>A p.(Asn4Lys)(N4K); deletion of the genes *GNAQ*, *ARID2*, *CDK4* and *MDM2*; deletion of the locus 12q12. **a** Low-power magnification of a nodular proliferation in the dermis. There is no epidermal melanocytic component. **b** Tumour displays a nested growth pattern. **c** Tumour cells with moderate nuclear atypia. Scattered melanophages are present within the tumour. **d** p16 immunohistochemistry reveals a somewhat checkerboard reaction pattern

Melanoma arising in a blue naevus (M-BN) is defined as a melanoma arising on the background of a blue naevus, most often a cellular blue naevus and less frequently from a common/dendritic blue naevus, or from different related intradermal melanocytic proliferations, including large plaque-type blue naevus and dermal melanocytoses ([1, 63, 64]; Fig. 9).

### Genetic landscape

The driver genetic aberrations in the development of melanoma arising in a blue naevus, featuring either mutations or gene fusions, are generally associated with dysregulation of the Gαq signalling pathway, leading to subsequent activation of the MAPK signalling pathway:

- *GNAQ* and *GNA11* somatic mutations, representing about 70% of driver genetic changes in blue naevi in total [65]. Of these, the *GNAQ* p.Q209 hotspot mutation is the most frequent genetic aberration, present in about 55% of blue naevi, followed by a GNAQ mutation in roughly 15% of blue naevi [63];
- *CYSLTR2* p.L12Q G‑coupled receptor gene mutation is a rare event, detected in about 3% of blue naevi [66];
- a phospholipase gene *PLCB4* hotspot activating mutation is exceptionally rare in cutaneous tumours, but it can be detected in about 3% of uveal melanomas [67];
- *protein kinase C* (*PRKC*) gene fusions represent a substantial proportion of the blue group of melanocytic proliferation, with *PRKCA *(alpha) gene fusion predominating over *PRKCB *(beta) gene fusions (ratio about 2:1) [68];
- *GRM1* fusion has only been detected in four cases to date [69].

After acquiring a driver gene aberration, malignant progression is generally achieved through acquisition of one of three mutually exclusive genetic changes in the genes *BAP1, SF3B1* and *EIF1AX *[70]. In addition, M‑BN typically features a few recurrent copy number alterations, including gains or amplifications of the 8q region (location of the protooncogene *c‑MYC*), isochromosome 6p, loss of 1p and monosomy 3 (linked to *BAP1* inactivation) [63]. Furthermore, *TERT* promoter mutations have been detected in about 50% of M‑BN [71, 72].

### Prognosis

The M‑BN variant of melanoma is aggressive with frequent metastases, in particular to the regional lymph nodes and liver [1]. In a literature review study analysing a total of 91 patients with M‑BN, metastatic spread was reported in 55% (*n* = 50) of the patients, with 16 patients presenting with metastatic disease, while another 16 patients developed dissemination within the first year and a further 18 patients within 5 years of the diagnosis [73]. Monosomy 3 and *BAP1* inactivation have been linked with an adverse prognosis [74].

## Practical recommendations for diagnostic molecular testing

Among individual genes, *TERT* promoter alteration and biallelic *CDKN2A* loss generally provide the most useful evidence of malignant progression, but both have limited sensitivity and context-dependent specificity. *PTEN* and *NF1* are more useful for pathway characterization than for resolving benignity versus malignancy. When the primary question is biological behaviour, an integrated assay that evaluates copy number complexity together with sequence variants and fusions is usually more informative. A negative ancillary test should not override convincing histopathological evidence of melanoma, and a positive test should not be interpreted without confirming adequate tumour content and clinicopathological concordance.

Molecular testing is most useful when it addresses a defined differential diagnosis after clinicopathological review and appropriate immunohistochemistry. No individual molecular alteration reliably separates all benign, dysplastic/intermediate and malignant melanocytic proliferations. Results should therefore be interpreted according to the pathways in the development of melanoma and be integrated with histopathology, clinical setting and follow-up. A practical approach is summarized in Table 2.

**Table 2:** Practical use of ancillary molecular tests in diagnostically challenging melanocytic lesions

Genetic pathway	Diagnostic setting	Suggested test strategy	Practical interpretation and limitations
I	Benign or dysplastic naevus vs. early conventional melanoma	PRAME immunohistochemistry as a first-line adjunct; if uncertainty remains, genome-wide copy number analysis (SNP array/aCGH or NGS copy number profiling) or melanoma FISH	Multiple or melanoma-associated copy number alterations support malignancy, whereas a flat profile supports a naevus but does not exclude melanoma. *BRAF* or *NRAS* mutations identify an initiating MAPK pathway driver and are not evidence of malignancy because they are common in naevi. *TERT* promoter mutation supports progression, particularly with additional aberrations, but its absence does not exclude melanoma
I	Deep-penetrating/WNT-activated lesion	Sequencing for combined MAPK pathway and WNT pathway activation (*CTNNB1* or *APC*), followed by copy number analysis and assessment of *CDKN2A* and the *TERT* promoter when malignant progression is suspected	The combined driver pattern establishes lineage. *TERT* promoter mutation, *CDKN2A* loss and multiple copy number alterations support WNT-activated melanoma but should not be used in isolation
I	*BAP1*-inactivated melanocytic tumour vs. *BAP1*-inactivated melanoma	*BAP1* and *BRAF-*V600E immunohistochemistry, with sequencing/copy number analysis when morphology suggests progression; consider germline evaluation in the appropriate personal or family setting	*BAP1* loss with *BRAF* or *NRAS* activation defines the pathway but does not establish melanoma. *TERT* promoter mutation and an increasingly complex copy number profile support malignant progression
II and III	High-CSD or desmoplastic melanoma and classification of an established melanoma	Broad DNA-based NGS when subtype assignment or therapeutic profiling is required; include *BRAF, NRAS, NF1* and *KIT*, and assess tumour mutational burden	*NF1* inactivation supports the high-CSD/desmoplastic molecular pathway but is not, by itself, a benign-versus-malignant discriminator. Likewise, *PTEN* loss is a progression event and potential pathway marker, particularly in *BRAF*-driven conventional melanoma, but is neither sufficiently sensitive nor specific for diagnosis
IV	Spitz naevus/atypical Spitz tumour vs. Spitz melanoma	DNA/RNA sequencing for kinase fusions or *HRAS*/*MAP3K8*/*MAP3K3* alterations to establish the Spitz pathway; copy number profiling, and targeted assessment of *CDKN2A* and the *TERT* promoter for risk stratification	A kinase fusion confirms pathway assignment, not malignancy. *CDKN2A* deletion, *TERT* promoter mutation and a complex copy-number profile favour malignant progression; isolated 11p gain with or without *HRAS* mutation favours Spitz naevus. Results must be integrated with morphology because no single alteration is fully sensitive
VIII	Blue naevus/atypical cellular blue tumour vs. melanoma arising in blue naevus	Sequencing for *GNAQ, GNA11, CYSLTR2, PLCB4* or relevant fusions to establish lineage; *BAP1* immunohistochemistry and genome-wide copy number profiling to evaluate progression	A Gαq-pathway driver is lineage defining rather than malignant. *BAP1 *loss, monosomy 3, 8q gain/amplification, additional progression-associated alterations and/or a complex copy-number profile support melanoma. *TERT* promoter mutation may add support but has incomplete sensitivity

*SNP* single-nucleotide polymorphism, *aCGH *array-based comparative genomic hybridization, *NGS *next-generation sequencing, *FISH* fluorescence in situ hybridisation, *CSD *cumulative sun damage

## Future perspectives

Advances in molecular diagnostics continue to expand the capabilities of genomic analysis in melanocytic tumours. Emerging technologies, including whole-exome sequencing and liquid biopsy approaches, are expected to further improve diagnostic accuracy and enable personalized treatment strategies. As knowledge of tumour biology continues to grow, molecular classification systems may become increasingly important in guiding clinical management. The integration of molecular diagnostics into the evaluation of melanocytic tumours has significantly improved the diagnostic accuracy and understanding of tumour biology.

## Practical conclusion

- Melanoma is not a single disease.
- A total of nine genetic pathways are recognized in melanoma development, leading to nine different melanoma subsets.
- It is crucial for pathologists to recognize melanoma subsets, since they differ significantly in biological behaviour and treatment.
- While histological examination supplemented by immunohistochemistry represents the gold standard for diagnosing melanoma, selected melanocytic tumours cannot be diagnosed without the aid of molecular genetic techniques.
- Spitz melanoma can only be diagnosed by molecular genetic techniques.
- Nodular melanoma represents a subset of melanoma in the vertical growth phase lacking a radial growth phase. This melanoma subtype can theoretically develop in the background of all genetic pathways with an endpoint of melanoma.
- Dedifferentiated melanoma is defined as a variant of melanoma featuring transitions between a conventional melanoma and dedifferentiated component, the former lacking histological and immunohistochemical features of melanocytic differentiation.
- Undifferentiated melanoma lacks a conventional melanoma component and can only be recognized as melanoma by molecular genetic features.

## Data Availability

All data supporting the findings of this work are included in the article.

## Declarations

The authors confirm that the paper complies with the relevant ethical guidelines.
